# The development of a combined mathematical model to forecast the incidence of hepatitis E in Shanghai, China

**DOI:** 10.1186/1471-2334-13-421

**Published:** 2013-09-08

**Authors:** Hong Ren, Jian Li, Zheng-An Yuan, Jia-Yu Hu, Yan Yu, Yi-Han Lu

**Affiliations:** 1 Department of Infectious Disease Control and Prevention, Shanghai Municipal Center for Disease Control and Prevention, Shanghai 200336, China; 2 The Key Laboratory of Public Health Safety of Minister of Education - Department of Epidemiology, Fudan University School of Public Health, Building 8 Room 441, 138 Yi Xue Yuan Road, Shanghai 200032, China; 3 Department of Injury Control and Prevention, Shanghai Municipal Center for Disease Control and Prevention, Building 1 Room 419, 1380 West Zhong Shan Road, Shanghai 200032, China

**Keywords:** Hepatitis E, Combined mathematical model, Forecast

## Abstract

**Background:**

Sporadic hepatitis E has become an important public health concern in China. Accurate forecasting of the incidence of hepatitis E is needed to better plan future medical needs. Few mathematical models can be used because hepatitis E morbidity data has both linear and nonlinear patterns. We developed a combined mathematical model using an autoregressive integrated moving average model (ARIMA) and a back propagation neural network (BPNN) to forecast the incidence of hepatitis E.

**Methods:**

The morbidity data of hepatitis E in Shanghai from 2000 to 2012 were retrieved from the China Information System for Disease Control and Prevention. The ARIMA-BPNN combined model was trained with 144 months of morbidity data from January 2000 to December 2011, validated with 12 months of data January 2012 to December 2012, and then employed to forecast hepatitis E incidence January 2013 to December 2013 in Shanghai. Residual analysis, Root Mean Square Error (RMSE), normalized Bayesian Information Criterion (BIC), and stationary R square methods were used to compare the goodness-of-fit among ARIMA models. The Bayesian regularization back-propagation algorithm was used to train the network. The mean error rate (MER) was used to assess the validity of the combined model.

**Results:**

A total of 7,489 hepatitis E cases was reported in Shanghai from 2000 to 2012. Goodness-of-fit (stationary R^2^=0.531, BIC= −4.768, Ljung-Box Q statistics=15.59, *P*=0.482) and parameter estimates were used to determine the best-fitting model as ARIMA (0,1,1)×(0,1,1)_12_. Predicted morbidity values in 2012 from best-fitting ARIMA model and actual morbidity data from 2000 to 2011 were used to further construct the combined model. The MER of the ARIMA model and the ARIMA-BPNN combined model were 0.250 and 0.176, respectively. The forecasted incidence of hepatitis E in 2013 was 0.095 to 0.372 per 100,000 population. There was a seasonal variation with a peak during January-March and a nadir during August-October.

**Conclusions:**

Time series analysis suggested a seasonal pattern of hepatitis E morbidity in Shanghai, China. An ARIMA-BPNN combined model was used to fit the linear and nonlinear patterns of time series data, and accurately forecast hepatitis E infections.

## Background

Hepatitis E is a liver disease caused by hepatitis E virus (HEV), a non-enveloped, positive-sense, single-stranded RNA virus which is transmitted mainly through contaminated drinking water or uncooked/undercooked food [1]. Since the earliest report of this water-borne disease in New Delhi, India during 1955 to 1956, it has been epidemic in many developing countries [2]. Every year there are 20 million hepatitis E infections, over 3 million acute cases of hepatitis E, and 70,000 hepatitis E-related deaths in the world. The prevalence is highest in Eastern and Southern Asia [3]. Sporadic hepatitis E has also become an important public health concern in developed countries, causing over 50% of acute viral hepatitis cases in recent years [4-7].

Shanghai is the largest metropolis in China with a permanent population of over 23.8 million. About 14 million are officially registered residents and 9.7 million are migrants. In order to control the spread of HEV, a surveillance system was established and a series of studies of HEV genotype, transmission route, and risk factors for infection have been conducted in Shanghai since 1997 [8-10]. According to surveillance data from Shanghai Municipal Center for Disease Control and Prevention, hepatitis E has been far more common than hepatitis A since 2004. Many researchers have developed mathematical models to forecast the incidence of hepatitis E.

Few mathematical models are applicable for modeling as time series data of hepatitis E infection has both linear and nonlinear characteristics. Autoregressive integrated moving average (ARIMA) has become one of the most popular and convenient linear models in time series forecasting [11-14]. It has advantages in both statistical properties and Box-Jenkins methodology in the model building process [15]. Although the ARIMA model could fit several different types of time series data, the major limitation is the pre-assumed linearity of the model [16]. In contrast, artificial neural networks (ANNs) have the ability to learn and describe highly-nonlinear and strongly-coupled relationships between multi-input and multi-output variables [17], and have no need to specify a detailed model. However, ANNs cannot handle both linear and nonlinear patterns equally well [18]. We designed a combined model using an ARIMA model and a neural network to forecast the incidence of hepatitis E in Shanghai.

## Methods

### Data source

Hepatitis E is one of Nationally Notifiable Infectious Diseases in China. Upon laboratory confirmation, hospital physicians register each patient’s information in the China Information System for Disease Control and Prevention within 24 hours. Community physicians then conduct an epidemiological investigation, health education, and three months follow-up of each patient and their family members. The morbidity data of hepatitis E from 2000 to 2012 were released from the China Information System for Disease Control and Prevention by Shanghai Municipal Center for Disease Control and Prevention. The annual average population data from 2000 to 2012 was obtained from Shanghai Public Security Bureau.

### The model

The ARIMA-BPNN combined model consisted of an ARIMA model and a back propagation artificial neural network (BPNN). The model was developed to forecast the incidence of hepatitis E in Shanghai. The model was trained using 144 months of morbidity data from January 2000 to December 2011, validated with 12 months of morbidity data from January 2012 to December 2012, and finally employed to forecast the incidence of hepatitis E from January 2013 to December 2013 in Shanghai. The whole process was divided into three steps:

The first step was to determine the best-fitting ARIMA model and to predict the values of each time point. The Box-Jenkins approach was applied to seasonal ARIMA (*p,d,q*)×(*P,D,Q*)_n_ modeling of time series data. The model was defined with an autoregressive part of order *p*, a moving average part of order *q*, a seasonal-autoregressive part of order *P*, a seasonal-moving average part of order *Q,* differencing and seasonal-differencing orders *d* and *D*, and periodic variable n. This model building process was designed to take advantage of associations in the seasonally and sequentially lagged relationships that usually exist in periodically collected data. Model parameters were estimated using the conditional Least Squares method. Residual analysis, Root Mean Square Error (RMSE), normalized Bayesian Information Criterion (BIC), and stationary R square were conducted to compare the goodness-of-fit among ARIMA models.

**Figure 1:**
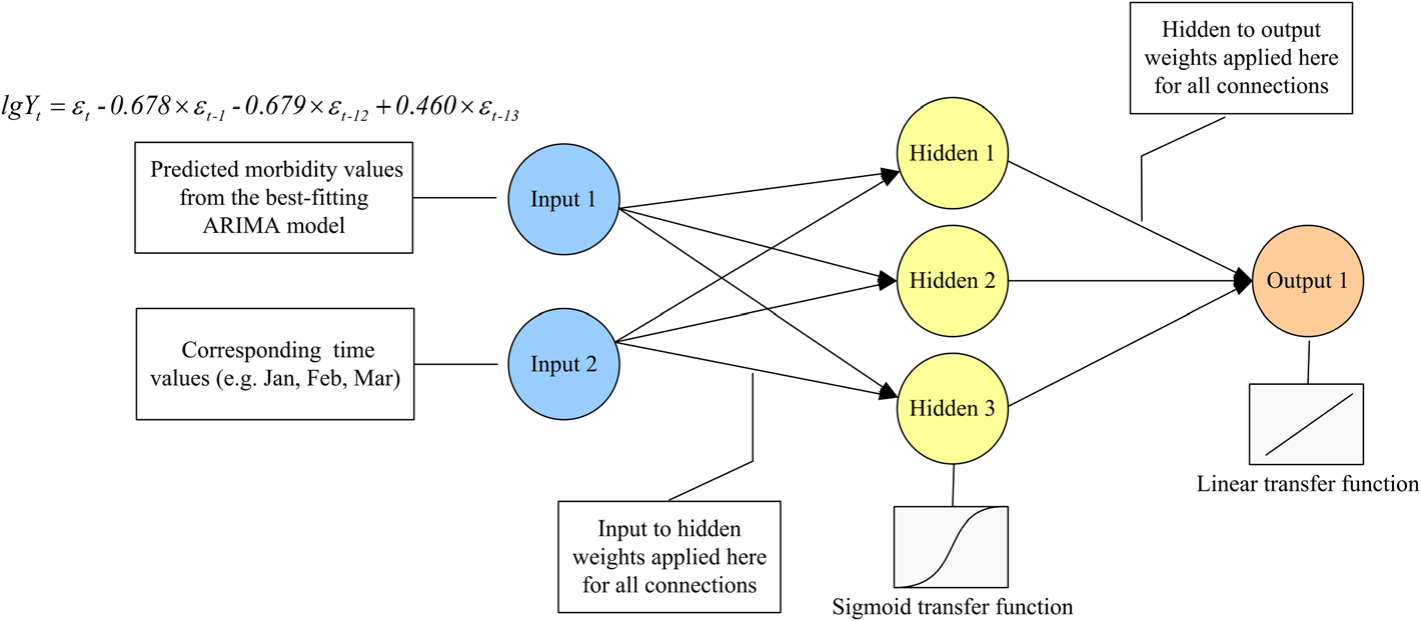
**The combination of ARIMA and BPNN models.** The ARIMA-BPNN combined model consisted of three layers: 2 neurons collected predicted morbidity values from ARIMA and corresponding time values in the input layer, 3 neurons estimated the actual morbidity values as targets and made a simulation in the hidden layer, and 1 neuron transferred the forecasted incidence to the output layer.

The second step was to train the BPNN. Neuron model and network architectures of BPNN have been previously reviewed [19]. In our study, the BPNN architecture consisted of three layers. Two neurons collected predicted morbidity values from ARIMA and corresponding time values in the input layer, 3 neurons estimated the actual morbidity values as targets and made a simulation in the hidden layer, and 1 neuron transferred the forecasted incidence to the output layer. The neurons in the hidden layer had a hyperbolic tangent sigmoid transfer function and the neuron in the output layer had a linear transfer function (Figure 1). A Bayesian regularization back-propagation algorithm was used to train the network and provide a unifying approach for dealing with issues of model complexity and over fitting [20].

The third step was to validate the combined model with 12 months of morbidity data from January 2012 to December 2012 and to further forecast the incidence of hepatitis E in 2013.

The mean error rate (MER) was used to explain the comparison of predicted and actual values between single ARIMA and ARIMA-BPNN combined models in 2012.

### Data processing and analysis

An augmented Dickey-Fuller test and the X-12-ARIMA seasonal adjustment program of Eviews 5.0 (http://www.eviews.com) were employed to determine the stabilization of time series data [21]. All analyses were performed using SPSS 17.0 (Chicago, IL, USA) and MATLAB 7.0 (Natick, USA).

### Ethical review

The study protocol and utilization of hepatitis E morbidity data were reviewed by Shanghai Municipal Center for Disease Control and Prevention and no ethical issues were identified. Therefore, no ethics approval was required by our Investigation Review Board.

## Results

### General patterns of hepatitis E

**Figure 2:**
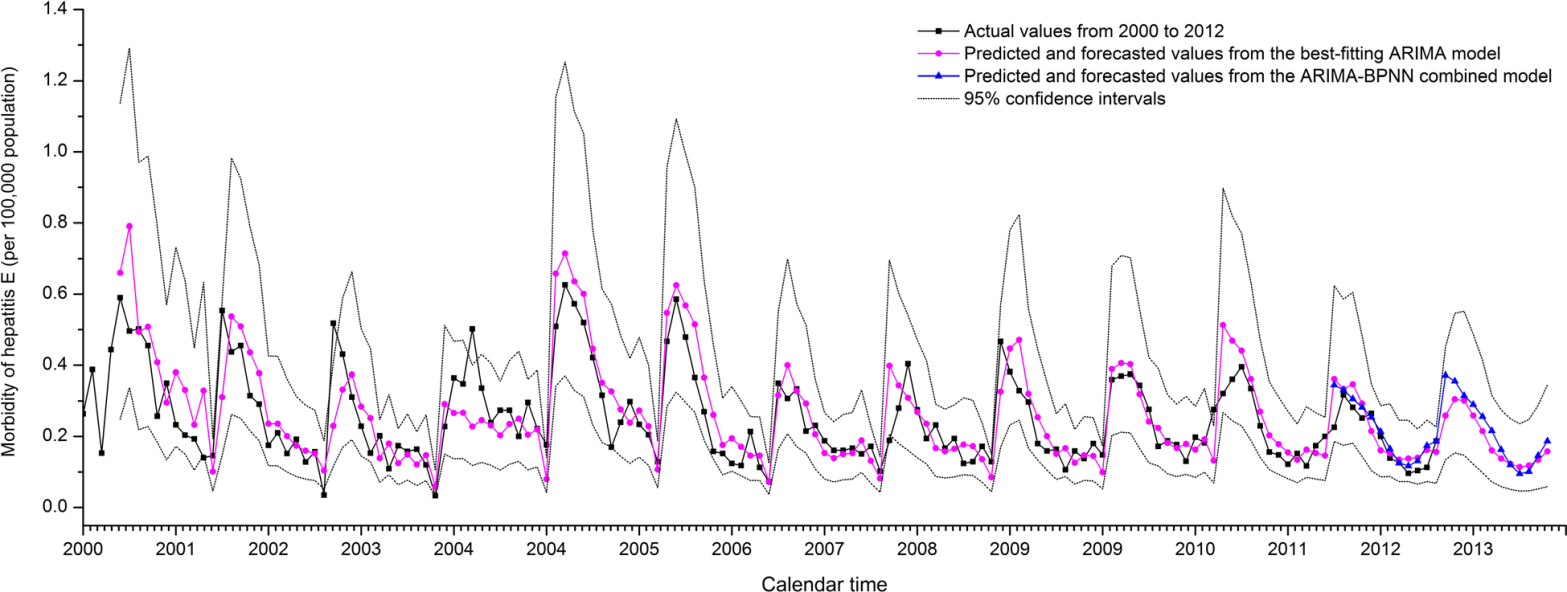
**Comparison of actual, predicted and forecasted morbidity rates of hepatitis E (2000–2013) in Shanghai, China.** The x-axis represents calendar time from 2000 to 2013. The y-axis represents actual morbidity rates and predicted/forecasted morbidity values of hepatitis E (per 100,000 population). From January 2001 to December 2012, morbidity values were predicted using the best-fitting ARIMA model or the ARIMA-BPNN model. From January 2013 to December 2013, morbidity values were forecasted using the best-fitting ARIMA model or the ARIMA-BPNN model. Forecast values for the two models were 0.259 and 0.372 (Jan), 0.305 and 0.356 (Feb), 0.301 and 0.315 (Mar), 0.259 and 0.290 (Apr), 0.215 and 0.256 (May), 0.161 and 0.216 (Jun), 0.138 and 0.163 (Jul), 0.123 and 0.120 (Aug), 0.114 and 0.095 (Sep), 0.118 and 0.101 (Oct), 0.134 and 0.146 (Nov), 0.158 and 0.187 (Dec), respectively. 95% confidence intervals are presented.

A total of 7,489 sporadic hepatitis E cases was reported in Shanghai from 2000 to 2012. This included registered residents and the immigrant population. The annual incidence rate declined to 2.307 per 100,000 population in 2012 and then fluctuated 2.307 to 4.240 per 100,000 population (Table 1). The male morbidity was significantly higher than that of females (*t*=8.951, *P*<0.001). The X-12-ARIMA seasonal adjustment program showed that the monthly morbidity data of hepatitis E from 2000 to 2012 had seasonal variations with a peak during January-March and a nadir from August-October (*F*=40.02, *P*<0.001) (Figure 2).

**Table 1:** The morbidity of hepatitis E in Shanghai from 2000 to 2012 (per 100,000 population)

**Year**	**Male**		**Female**		**Total**	
	**Cases**	Morbidity*	**Cases**	Morbidity*	**Cases**	**Morbidity**
		**(per 100,000 pop.)**		**(per 100 000 pop.)**		**(per 100,000 pop.)**
2000	498	7.507	224	3.425	722	4.240
2001	465	6.972	222	3.377	687	4.010
2002	354	5.282	178	2.695	532	3.100
2003	312	4.630	165	2.484	477	2.600
2004	425	6.269	182	2.720	607	3.460
2005	505	7.405	220	3.263	725	4.240
2006	387	5.649	167	2.459	554	3.120
2007	299	4.340	173	2.527	472	2.540
2008	305	4.475	157	2.318	462	2.490
2009	345	5.027	166	2.424	511	2.710
2010	364	5.282	221	3.201	585	3.050
2011	391	5.584	233	3.309	624	2.711
2012	313	4.470	218	3.096	531	2.307

*Represents the morbidity of registered residents in Shanghai.

### The best-fitting ARIMA model

Since the time series data of hepatitis E morbidity had both seasonal and non-seasonal trends, a logarithmic transformation (non-seasonal and seasonal first order differencing) were employed to stabilize the series (Augmented Dickey-Fuller test: *t*= −13.23, *P*<0.001). The goodness-of-fit (stationary R^2^=0.531, RMSE= 0.084, BIC= −4.768, Ljung-Box Q statistics=15.59, *P*=0.482) and parameter estimates (Table 2) determined the best-fitting ARIMA model to be ARIMA (0,1,1)×(0,1,1)_12_. The equation was lg *Y*_t_ = *ϵ*_t_ ‒ 0.678 × *ϵ*_t ‒ 1_ ‒ 0.679 × *ϵ*_t ‒ 12_ + 0.460 × *ϵ*_t ‒ 13_.

**Table 2:** **Parameters for the final seasonal ARIMA (0,1,1)×(0,1,1)**_**12**_** model**

**Parameter**	**Estimation**	**Standard error**	***t *statistics**	***P *Value**
Constant	0.000	0.000	−0.113	0.911
MA_1_	0.678	0.067	10.12	0.000
SMA_1_	0.679	0.093	7.318	0.000

*ARIMA*, Autoregressive integrated moving average model.

The predicted values from best-fitting ARIMA model in 2012 fluctuated from 0.135 to 0.362 per 100,000 population, with the same seasonal variation as the actual ones. The MER of the best-fitting ARIMA model was 0.250 (Table 3, Figure 2).

**Table 3:** Predicted and error rates of the single ARIMA model and ARIMA-BPNN combined model in 2012

**Month**	**Morbidity (per 100,000 pop.)**	**ARIMA model**		**ARIMA-BPNN model**	
		**Predicted rate**	**Error rate**	**Predicted rate**	**Error rate**
Jan	0.226	0.362	0.602	0.345	0.527
Feb	0.317	0.334	0.054	0.331	0.044
Mar	0.282	0.347	0.230	0.306	0.085
Apr	0.252	0.293	0.163	0.282	0.119
May	0.265	0.215	0.189	0.254	0.042
Jun	0.200	0.161	0.195	0.214	0.070
Jul	0.139	0.152	0.094	0.165	0.187
Aug	0.126	0.135	0.071	0.126	0.000
Sep	0.096	0.138	0.438	0.117	0.219
Oct	0.104	0.140	0.346	0.131	0.260
Nov	0.113	0.162	0.434	0.174	0.540
Dec	0.191	0.156	0.183	0.187	0.021
MER			0.250		0.176

*ARIMA*, Autoregressive integrated moving average model; *BPNN*, Back propagation neural network; *MER*, Mean error rate.

### ARIMA-BPNN combined model

To construct the ARIMA-BPNN combined model, the predicted morbidity values from the best-fitting ARIMA model and corresponding time values were used as input (2×131 matrix), while the actual morbidity values were used as target data (1×131 matrix) (Figure 1). The model fitted values in 2012 fluctuated from 0.117 to 0.345 per 100,000 population. The MER of the ARIMA-BPNN combined model was 0.176, lower than the 0.250 MER of the single ARIMA model. This proved that the combined model was more effective.

The combined model was then used to forecast the incidence of hepatitis E in 2013. The prediction was a continued fluctuance within a narrow range from 0.095 to 0.372 per 100,000 population, with a peak during winter (January-March) and a nadir during autumn (August-October) (Figure 2).

## Discussion

Hepatitis E is generally regarded as a disease predominantly restricted to areas with poor sanitation and polluted drinking water supplies [22]. However, more cases due to zoonotic spread and unclear transmission methods are occurring in non-endemic areas including Shanghai, China [10,23,24]. A total of 7,489 hepatitis E cases was reported in Shanghai from 2000 to 2012. The incidence fluctuated between 2.307 and 4.240 per 100,000 population, with seasonal variations. This has led to a major shift in the understanding of the epidemiology of hepatitis E and warranted further study.

Compared to blood-borne infectious diseases (e.g. hepatitis B and C, AIDS), hepatitis E is more affected by environmental and natural factors. These factors lead to a seasonal variation in incidence. The multiple factors involved cause difficulties in modeling. Time series analysis has the advantage of forecasting the incidence without focusing on specific risk factors; however, it cannot describe a nonlinear trend in incidence data. ANNs have been widely accepted as a potentially useful means in modeling complex nonlinear and dynamic systems which could remove the need for model builders to correctly specify the precise functional forms of the relationship that the model seeks to represent. However, they still require the need for knowledge as well as prior information about the systems of interest [25-27]. It has been argued that combining multiple models for forecasting may provide better estimates than single time series models, by taking advantage of each model’s capabilities [18,28]. Accordingly, we constructed a hybrid architecture which comprised an ARIMA model and a neural network for forecasting hepatitis E incidence and validated its efficacy. The MER of the single ARIMA model and the ARIMA-BPNN combined model were 0.250 and 0.176, respectively. The combined model forecasted that the incidence of hepatitis E in Shanghai in 2013 would be similar to that of previous years, and that there would be a seasonal variation with a peak during winter and a nadir during autumn.

We determined that an ARIMA-BPNN combined model better fit time series data of hepatitis E morbidity in Shanghai than a single ARIMA model. This combined method could not be applied to all time series data without assuming that the relationship between the linear and non-linear components was additive. If the relationship was different (e.g. multiplicative), the combined method would lower the capacity [29]. The morbidity of hepatitis E was influenced by many environmental and natural factors which are dynamic and possibly evolving over time. Thus, the parameters of an ARIMA-BPNN combined model should be periodically re-assessed according to continuously updated data to maintain long-term sustainability and precision.

## Conclusions

Time series analysis demonstrated a seasonal pattern of hepatitis E infection in Shanghai, China. An ARIMA-BPNN combined model was used to describe the linear and nonlinear patterns of the time series data. This model effectively forecasts hepatitis E infection. We focused on the ARIMA-BPNN combined model because single ARIMA and BPNN models had been intensively studied. The construction and interpretation of other combined analyses should be explored.

## Abbreviations

ARIMA: Autoregressive integrated moving average model; BPNN: Back propagation neural network; BIC: Bayesian information criterion; MER: Mean error rate; HEV: Hepatitis E virus; RMSE: Root mean square error.

## Competing interests

The authors declare that they have no competing interests.

## Authors’ contributions

HR, YY and YHL conceived the study, performed the statistical analysis and drafted the manuscript. JL, ZAY and JYH assisted with data collection and statistical analysis. All authors contributed to the interpretation of the data and the preparation of the manuscript. All authors read approved the final manuscript.

## Pre-publication history

The pre-publication history for this paper can be accessed here:

http://www.biomedcentral.com/1471-2334/13/421/prepub
